# Shorter perioperative QRS duration and a leftward frontal-plane QRS axis are associated with anastomotic leakage after esophagectomy: a hypothesis-generating study

**DOI:** 10.3389/fmed.2026.1942699

**Published:** 2026-09-29

**Authors:** Mengke Ma, Youjia Zhu, Handan Lin, Weizhuo Liu, Wendi Fei, Haotian Jiang, Bin He

**Affiliations:** Department of Critical Care Medicine and Emergency, Shanghai Chest Hospital, Shanghai Jiao Tong University School of Medicine, Shanghai, China

**Keywords:** anastomotic leakage, electrocardiography, esophageal cancer, esophagectomy, perioperative monitoring, QRS axis, QRS duration

## Abstract

**Background:**

Anastomotic leakage (AL) after esophagectomy is a serious complication for which early physiological signals remain limited. We conducted a hypothesis-generating analysis of whether routinely reported electrocardiographic QRS measurements were associated with AL during the perioperative period.

**Methods:**

This single-center retrospective cohort included 292 of 1,441 patients undergoing esophagectomy who had an index 12-lead electrocardiogram (ECG) from day −1 through postoperative day 1. ECG acquisition followed routine clinical practice rather than a study protocol. QRS duration and frontal-plane QRS axis were retained after descriptive screening of the continuous ECG measurements available in the analytic extract. They were entered into a two-variable logistic model, followed by a sensitivity model additionally adjusting for seven clinical characteristics. Timing-stratified associations and same-cohort model discrimination were also examined.

**Results:**

AL occurred in 29 of 292 patients (9.9%). Patients with AL had a shorter QRS duration (median, 86 vs. 92 ms; *P* = 0.016) and a more leftward QRS axis (4 degrees vs. 27 degrees; *P* = 0.006). In the two-variable model, standardized odds ratios (ORs) were 0.378 (95% CI, 0.178-0.801) for QRS duration and 0.501 (0.323-0.779) for QRS axis. In the sensitivity model, the corresponding ORs were 0.359 (0.170-0.761) and 0.486 (0.299-0.789). The QRS-axis association was stronger preoperatively, whereas the QRS-duration association was stronger from day 0 through postoperative day 1. Median repeated-cross-validation AUCs were 0.702 for the ECG model, 0.559 for the clinical reference model, and 0.690 for the combined model; bootstrap confidence intervals for both AUC differences versus the clinical reference included zero.

**Conclusion:**

Shorter QRS duration and a more leftward frontal-plane QRS axis were associated with AL after esophagectomy. These QRS measurements are potential perioperative signals that require prospective validation rather than established predictors.

## 1 Introduction

Esophageal cancer remains a major global health burden (1, 2). Esophagectomy using open, minimally invasive, or hybrid approaches remains central to curative-intent treatment for resectable disease, but its benefits are accompanied by substantial perioperative morbidity (3, 4). Anastomotic leakage (AL) and conduit necrosis are among the most consequential complications (5–7). They can prolong hospitalization, delay subsequent treatment, necessitate endoscopic or surgical intervention, and increase the risk of death. Early recognition is therefore a central objective of postoperative surveillance (8).

Clinical suspicion of AL is usually assembled from symptoms, bedside observations, laboratory trends, drain findings, and imaging. Postoperative inflammatory biomarkers and systemic inflammatory responses have been evaluated as aids for detecting or anticipating AL, while multimodal models combining clinical and imaging data are also under investigation (9–12). Beyond AL-specific surveillance, perioperative hemodynamic prediction and surgical-oncology biomarker studies illustrate the broader translational interest in low-burden signals available during routine care (13, 14). These approaches remain indispensable, but many are intermittent, require additional testing, or become informative only after a systemic response is established. A physiological signal that is already acquired in routine care, automatically quantified, and available without additional sampling could therefore provide complementary information.

The 12-lead electrocardiogram (ECG) contains quantitative information beyond rhythm diagnosis alone. QRS duration describes the time required for ventricular depolarization, whereas frontal-plane QRS axis summarizes the net direction of ventricular electrical activation in the frontal plane (15, 16). Both measurements can vary with conduction phenotype, cardiac orientation, thoracic anatomy, loading conditions, and broader physiological state (17, 18). Because frontal QRS axis is influenced by cardiac orientation and body build, while gastric conduit reconstruction places an anastomosis within or across the thoracic and mediastinal anatomy, we considered whether these routinely reported measurements might capture anatomical or physiological context associated with AL. This rationale was hypothesis-generating and did not assume that QRS measurements directly alter anastomotic pressure or tension. ECG-derived measures have supported perioperative cardiovascular risk assessment after noncardiac surgery, and ECG-based physiological estimates have shown prognostic value in other surgical populations (19–21). Perioperative vital-sign dynamics have also been investigated as early markers of infectious complications, providing an adjacent, although not ECG-specific, signal-monitoring precedent (22). However, the relationship between standard QRS measurements and AL after esophagectomy has not been established.

The timing of ECG acquisition is important when interpreting such a relationship. A day −1 ECG clearly precedes surgery and may characterize baseline susceptibility. A recording obtained on the surgery day or postoperative day 1 may reflect baseline features, operative stress, fluid shifts, inflammation, autonomic responses, or an evolving postoperative complication. Pooling these time points addresses whether a perioperative ECG signal is associated with AL in routine practice, but timing-specific analyses are needed to distinguish baseline association from a potential perioperative or early monitoring signal.

We therefore conducted a hypothesis-generating study of the relationship between perioperative QRS duration, frontal-plane QRS axis, and AL. We first quantified the pooled perioperative associations, then examined the preoperative and day-0-to-postoperative-day-1 periods separately. We further compared discrimination of a two-variable ECG model with a clinical reference model and a combined clinical-plus-ECG sensitivity model in the same patients. These analyses evaluated the strength and robustness of the observed ECG associations without treating the models as a validated clinical prediction system.

## 2 Materials and methods

### 2.1 Study design and cohort

This single-center retrospective cohort study was conducted at Shanghai Chest Hospital, a tertiary referral center for esophageal cancer surgery. Consecutive patients undergoing curative esophagectomy between January 2023 and December 2025 were identified from the institutional surgical database. The study was reported according to STROBE, with relevant TRIPOD principles used for the model-comparison analyses (23, 24).

The study protocol was approved by the Ethics Committee of Shanghai Chest Hospital on 4 June 2026 (approval no. IS26098). The requirement for individual informed consent was waived because of the retrospective design. The study was conducted in accordance with the Declaration of Helsinki.

The source cohort comprised 1,441 patients who underwent curative esophagectomy during the study period. Of these, 162 had no ECG record. Among the 1,279 patients with at least one ECG, 970 had no recording within the prespecified day −1-to-postoperative-day-1 window. Of the 309 patients with an ECG in that window, 17 lacked available QRS duration and frontal-plane QRS axis measurements. The remaining 292 patients (20.3%) had an eligible index ECG, and all had complete values for the retained ECG and clinical model variables; no further patients were excluded because of missing analysis variables. No values were imputed. Figure 1 summarizes the experimental workflow and participant selection, and the same 292-patient cohort was used for the association, model-comparison, and descriptive criterion analyses.

**Figure 1:**
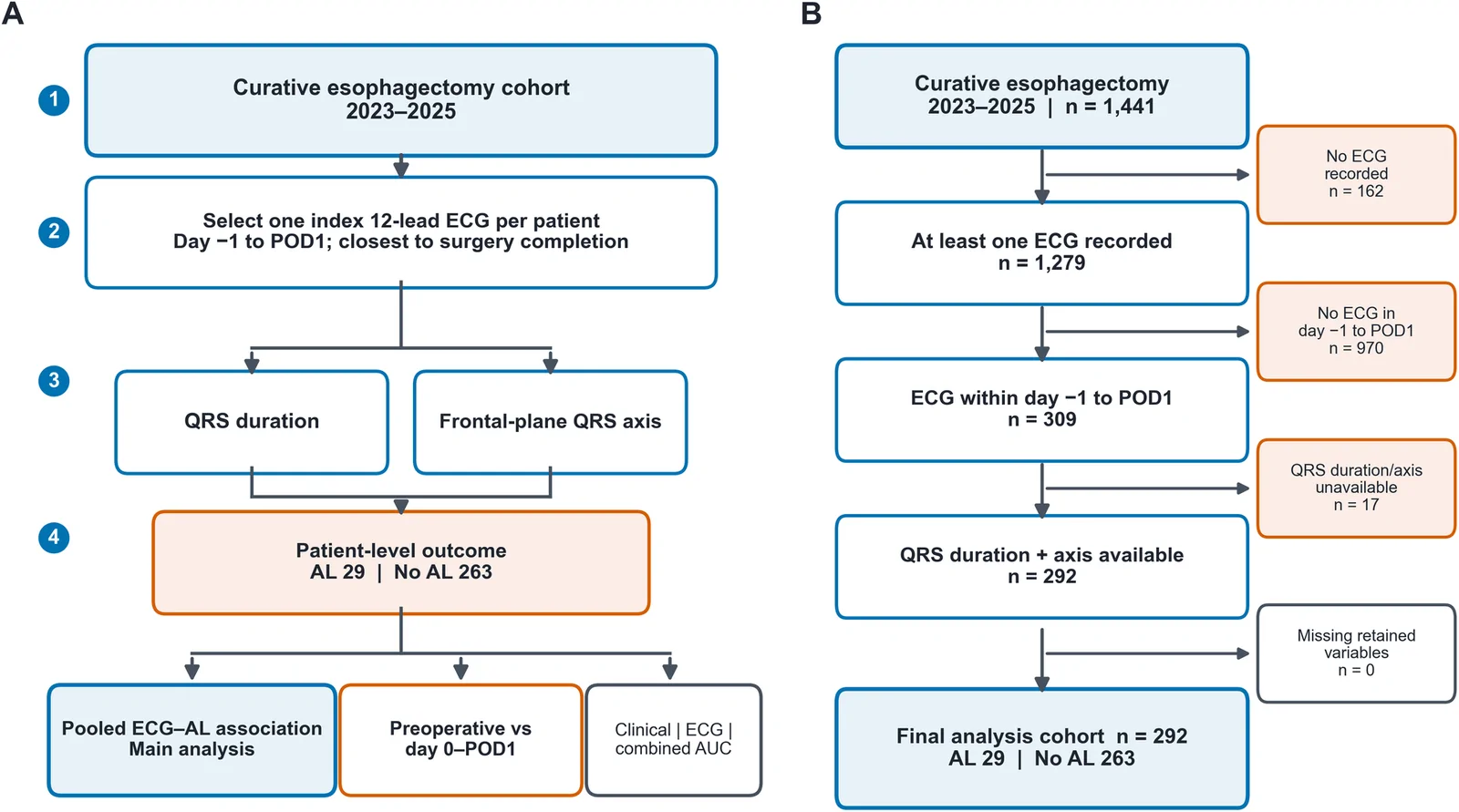
Study design and participant selection. **(A)** Experimental workflow. Consecutive patients undergoing curative esophagectomy from 2023 through 2025 were assessed for an eligible index 12-lead ECG from preoperative day −1 through postoperative day 1. Each patient contributed the eligible recording closest to surgery completion. QRS duration and frontal-plane QRS axis were related to patient-level AL status in the pooled main analysis; timing-stratified analyses compared preoperative with day-0-to-postoperative-day-1 ECGs, and model comparisons provided supporting discrimination analyses. **(B)** Participant selection. Among 1,441 surgical patients, 162 had no ECG record and 970 had no ECG within the prespecified window. Among 309 patients with an in-window ECG, 17 lacked the required QRS duration and frontal-plane QRS axis measurements. The remaining 292 patients had an eligible index ECG and complete retained analysis variables; none were excluded for missing retained variables. The final cohort included 29 patients with AL and 263 without AL. AL, anastomotic leakage; ECG, electrocardiogram; POD1, postoperative day 1.

### 2.2 Outcome and ECG measurements

The primary outcome was AL after esophagectomy, defined as disruption of the esophagogastric or esophagojejunal anastomosis supported by clinical, radiological, endoscopic, or operative evidence (25). Diagnoses recorded by the treating surgical team were verified by chart review. The outcome was analyzed as a binary patient-level endpoint. The analytic extract did not retain the date and time of first clinical suspicion or confirmed AL diagnosis; consequently, the postoperative day of diagnosis and whether an individual index ECG followed documented suspicion or diagnosis could not be determined retrospectively.

ECG acquisition was not protocol-driven and was performed according to routine perioperative clinical practice; availability therefore depended on clinical workflow rather than research enrollment. Standard 12-lead ECGs were recorded at a paper speed of 25 mm/s and gain of 10 mm/mV. QRS duration and frontal-plane QRS axis were extracted from the structured automated quantitative ECG report and were not manually remeasured for this study. The de-identified analytic export did not retain the ECG manufacturer, device model, or algorithm/software version. It also did not retain sufficiently detailed diagnostic labels to apply a reproducible exclusion for bundle branch block, ventricular pacing, or other major conduction abnormalities; therefore, no separate conduction-based exclusion was applied, and all eligible reports with available QRS duration and axis were retained. For each patient, the index ECG was the eligible recording closest to surgery completion, so that each participant contributed one ECG to the pooled analysis. Recordings were categorized by calendar day as day −1, the surgery day, or postoperative day 1. The export did not preserve clock time relative to incision or surgery completion, so surgery-day ECGs could not be classified reliably as preoperative or postoperative. For the timing analysis, day −1 represented the preoperative period, while the surgery day and postoperative day 1 were combined as the day-0-to-postoperative-day-1 period because postoperative day 1 alone contained only one AL event.

The auditable analytic extract contained three continuous routinely reported ECG measurements for screening: heart rate, QRS duration, and frontal-plane QRS axis. Their distributions were compared between patients with and without AL in the full analysis cohort. QRS duration and frontal-plane QRS axis were retained through this data-driven screening and were not prespecified before inspection of the data. The complete screening results are reported in Supplementary Table S3. Screening *P* values were descriptive and were not adjusted for multiplicity. Continuous values were used for the principal association models to avoid loss of information from dichotomization. Two same-cohort criteria, QRS duration ≤92 ms and QRS axis ≤20°, were additionally derived from full-cohort Youden indices (26). They were used only to visualize the observed gradient in AL proportions according to the number of criteria met; they were not treated as validated clinical thresholds.

### 2.3 Clinical reference and model assessment

The clinical reference model included age, sex, body mass index, diabetes, smoking history, operation duration, and anastomosis site. Age, sex, and body mass index represented general baseline characteristics; diabetes and smoking represented routinely available metabolic and vascular risk factors; and operation duration and anastomosis site represented procedural context directly relevant to esophagectomy. These seven variables were chosen because of their clinical relevance and complete availability in the analysis file. The clinical reference model placed the ECG associations in conventional patient and procedural context. A combined clinical-plus-ECG model added QRS duration and frontal-plane QRS axis and served both as a confounder-adjusted sensitivity analysis of the ECG coefficients and as an incremental discrimination comparison.

All three models used the same 292 participants and 29 AL events, allowing direct comparison on an identical analysis set. Because the event count was limited relative to the seven- and nine-variable models, their coefficients and performance estimates were interpreted cautiously.

### 2.4 Statistical analysis

Continuous variables were summarized as medians and interquartile ranges (IQRs) and compared using two-sided Mann–Whitney U tests because the analysis did not assume normally distributed measurements. Categorical variables were summarized as counts and percentages and compared using two-sided Fisher exact tests. All tests were two-sided. The screening family comprised heart rate, QRS duration, and frontal-plane QRS axis; screening *P* values were descriptive because the variables were examined in the same cohort without multiplicity adjustment.

The primary ECG association model was an unpenalized logistic regression containing standardized QRS duration and standardized frontal-plane QRS axis. Standardization placed the two measurements on comparable scales. As a sensitivity analysis, the combined model additionally adjusted for age, sex, body mass index, diabetes, smoking history, operation duration, and anastomosis site. Model coefficients, standard errors, intercepts, ORs, and Wald 95% CIs were estimated; ORs for continuous variables represent a one-standard-deviation increase. The seven-variable clinical reference model and the nine-variable sensitivity model were also used for comparative discrimination. Given 29 AL events, the larger models were not interpreted as stable prediction models (27, 28).

Discrimination was summarized by the area under the receiver operating characteristic curve (AUC). Apparent AUCs described performance when each model was fitted and evaluated in the complete analysis cohort. Internal validation used stratified five-fold cross-validation repeated across seeds 0 through 99 (29). Standardization and model fitting were repeated within each training fold, and predictions were generated only for the corresponding held-out fold. The median of the 500 held-out-fold AUCs was reported. Their 2.5th and 97.5th percentiles were shown as empirical fold intervals; because folds within repeated cross-validation are dependent, these intervals were not interpreted as confidence intervals.

Comparative uncertainty was assessed with 1,000 patient-level out-of-bag bootstrap resamples (30). Each model was fitted in a bootstrap sample and evaluated among participants not selected into that sample. Within each resample, AUC differences were calculated on the same out-of-bag participants. Percentile 95% CIs were then calculated for ECG minus clinical AUC and combined minus clinical AUC. This patient-level procedure preserved the paired nature of each comparison.

Timing analyses fitted the same two-variable ECG model separately in the preoperative and combined day-0-to-postoperative-day-1 groups, using full-cohort standard deviations to retain a common OR scale. Likelihood-ratio tests compared models with and without QRS-by-timing and axis-by-timing interaction terms. These tests assessed evidence that an association differed between periods; they did not test whether a measurement was associated with AL overall. Postoperative day 1 was not modeled separately because it contained one AL event. Observed AL proportions according to the number of ECG criteria met were reported with Wilson 95% CIs (31).

## 3 Results

### 3.1 Cohort characteristics

Figure 1B shows the sequential screening of the 1,441-patient surgical source cohort. A total of 162 patients had no ECG record, 970 had no ECG within the prespecified perioperative window, and 17 had an in-window ECG without the required QRS measurements. The remaining 292 patients (20.3%) had an eligible index ECG and complete retained analysis variables and entered the analysis. AL occurred in 29 participants (9.9%); the remaining 263 participants did not develop AL. Table 1 summarizes the analyzed cohort. Supplementary Table S1 shows that, compared with the 1,149 patients without an eligible ECG, included patients were older, more often current or former smokers, and more often underwent cervical anastomosis, whereas the observed AL proportion did not differ statistically between groups.

**Table 1:** Clinical characteristics and index-ECG timing.

Characteristic	Overall (*n* = 292)	AL (*n* = 29)	No AL (*n* = 263)	*P* value
Age, years	69.0 (63.0–74.2)	70.0 (61.0–75.0)	69.0 (63.0–74.0)	0.881
Body mass index, kg/m^2^	22.7 (20.5–24.8)	24.4 (21.0–25.5)	22.6 (20.4–24.6)	0.090
Operation duration, min	258.5 (222.8–302.2)	300.0 (237.0–322.0)	255.0 (220.0–300.0)	0.027
Male sex, *n* (%)	250 (85.6)	26 (89.7)	224 (85.2)	0.780
Diabetes mellitus, *n* (%)	44 (15.1)	6 (20.7)	38 (14.4)	0.410
Current or former smoker, *n* (%)	51 (17.5)	7 (24.1)	44 (16.7)	0.309
Cervical anastomosis, *n* (%)	186 (63.7)	15 (51.7)	171 (65.0)	0.161
Index ECG timing, *n* (%)				—
Day −1	115 (39.4)	11 (37.9)	104 (39.5)	
Surgery day	154 (52.7)	17 (58.6)	137 (52.1)	
Postoperative day 1	23 (7.9)	1 (3.4)	22 (8.4)	

Values are median (IQR) unless otherwise stated. Continuous variables were compared with two-sided Mann–Whitney U tests and binary variables with two-sided Fisher exact tests. No timing-distribution *P* value is reported because postoperative day 1 contained one AL event. AL, anastomotic leakage; ECG, electrocardiogram.

The cohort had a median age of 69 years, 250 participants (85.6%) were male, and 186 (63.7%) underwent cervical anastomosis. Operation duration was longer in participants with AL than in those without AL: 300 min (IQR, 237–322) versus 255 min (220–300; *P* = 0.027). For body mass index, age, sex, diabetes, smoking history, and anastomosis site, the univariable comparisons did not provide statistical evidence of between-group differences. The distribution of index-ECG timing is provided in Table 1.

### 3.2 Perioperative ECG associations

Figures 2A,B show the central ECG findings. Participants with AL had a shorter QRS duration than those without AL: 86 ms (IQR, 80–92) versus 92 ms (86–100; *P* = 0.016). Their frontal-plane QRS axis was also more leftward: 4° (IQR, −26° to 28°) versus 27° (7° to 47°; *P* = 0.006).

**Figure 2:**
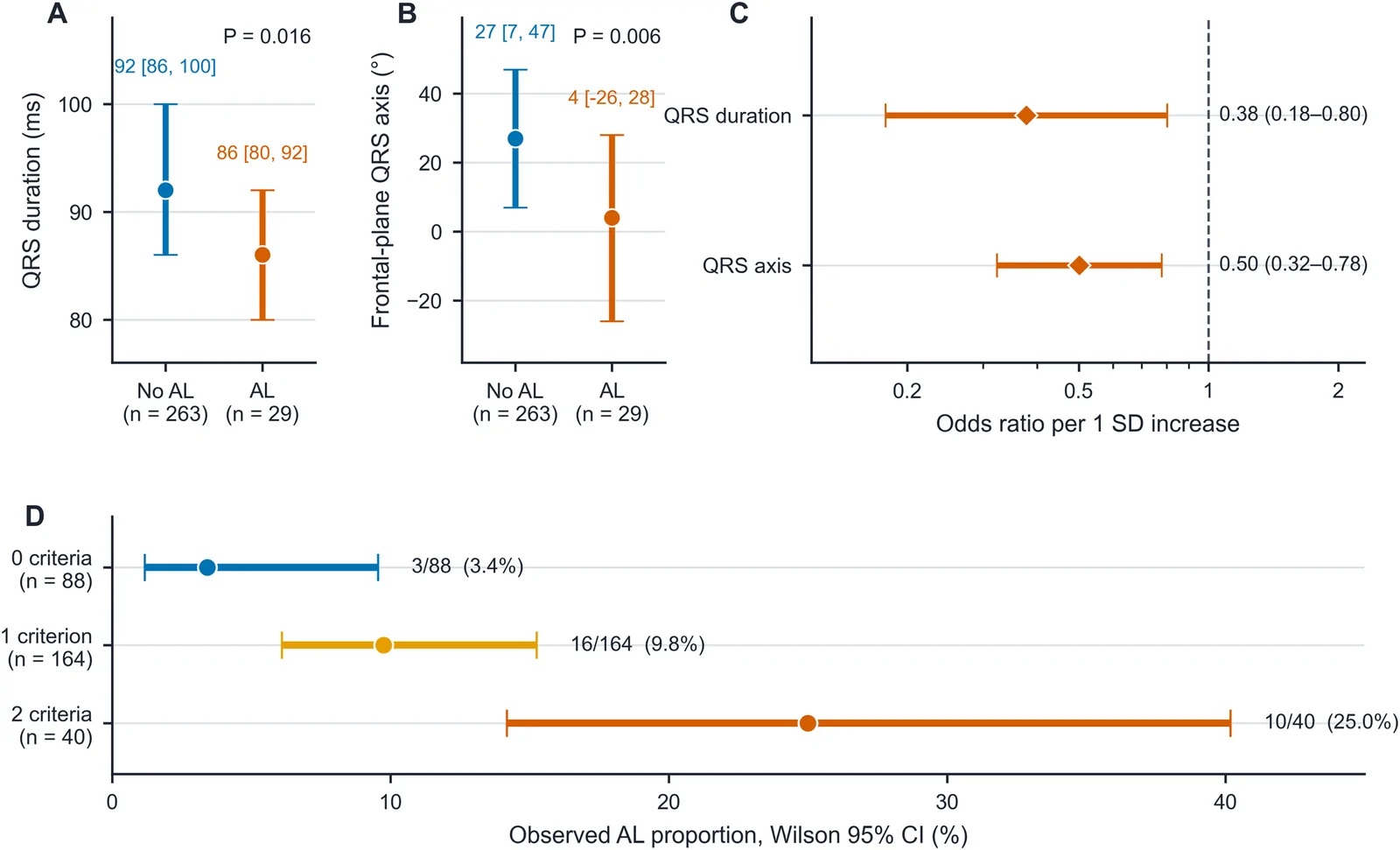
ECG associations with anastomotic leakage. **(A)** QRS duration and **(B)** frontal-plane QRS axis in participants with and without AL. Points show medians, whiskers show IQRs, and *P* values are from two-sided Mann–Whitney U tests. **(C)** Standardized ORs and Wald 95% CIs from the two-variable ECG model, in which QRS duration and QRS axis were mutually adjusted for each other without clinical covariates; ORs represent a one-SD increase. **(D)** Observed AL proportions and Wilson 95% CIs according to the number of data-derived ECG criteria met: QRS duration ≤92 ms and frontal-plane QRS axis ≤20°. ECGs from day −1 through postoperative day 1 were pooled in all four panels. AL, anastomotic leakage; CI, confidence interval; ECG, electrocardiogram; IQR, interquartile range; OR, odds ratio; SD, standard deviation.

In the two-variable ECG model, QRS duration and frontal-plane QRS axis were mutually adjusted for each other. A one-standard-deviation increase in QRS duration was associated with an OR of 0.378 (95% CI, 0.178-0.801; *P* = 0.011), and the corresponding OR for frontal-plane QRS axis was 0.501 (95% CI, 0.323-0.779; *P* = 0.002). Figure 2C displays these conditional estimates, and Table 2 reports the model coefficient, intercept, OR, and 95% CI. In the sensitivity model additionally adjusting for all seven clinical variables, the corresponding ORs remained below one: 0.359 (95% CI, 0.170-0.761; *P* = 0.007) for QRS duration and 0.486 (95% CI, 0.299-0.789; *P* = 0.004) for QRS axis. Full sensitivity-model coefficients are provided in Supplementary Table S2.

**Table 2:** Standardized ECG association model.

Term	Mean (SD)	Coefficient (SE)	OR (Wald 95% CI)	*P* value
Intercept	—	−2.567 (0.270)	—	<0.001
QRS duration	97.390 (16.708) ms	−0.974 (0.384)	0.378 (0.178–0.801)	0.011
Frontal-plane QRS axis	29.205 (37.488)°	−0.690 (0.225)	0.501 (0.323–0.779)	0.002

The model included 292 participants and 29 AL events. QRS duration and frontal-plane QRS axis were mutually adjusted for each other; no clinical covariates were included. Continuous-variable ORs represent a one-SD increase. AL, anastomotic leakage; CI, confidence interval; OR, odds ratio; SD, standard deviation; SE, standard error.

The observed AL proportion increased with the number of ECG criteria met, as illustrated in Figure 2D. AL occurred in 3 of 88 participants meeting neither criterion (3.4%; Wilson 95% CI, 1.2–9.5%), 16 of 164 meeting one criterion (9.8%; 6.1–15.3%), and 10 of 40 meeting both criteria (25.0%; 14.2–40.2%). Thus, the observed proportion was approximately threefold higher with one criterion than with neither criterion and was highest when both were present. Because both cut points and proportions came from the same cohort, this gradient is a descriptive representation of the continuous associations rather than an externally validated classification rule.

### 3.3 Timing patterns and model comparison

The timing-stratified analyses yielded estimates of different magnitudes across the perioperative periods. Preoperatively, the standardized OR was 0.276 (95% CI, 0.120–0.636) for frontal-plane QRS axis and 0.652 (95% CI, 0.225–1.892) for QRS duration. From day 0 through postoperative day 1, the corresponding ORs were 0.674 (95% CI, 0.384–1.182) for QRS axis and 0.285 (95% CI, 0.106–0.763) for QRS duration. Figures 3A,B place these timing-specific estimates side by side.

**Figure 3:**
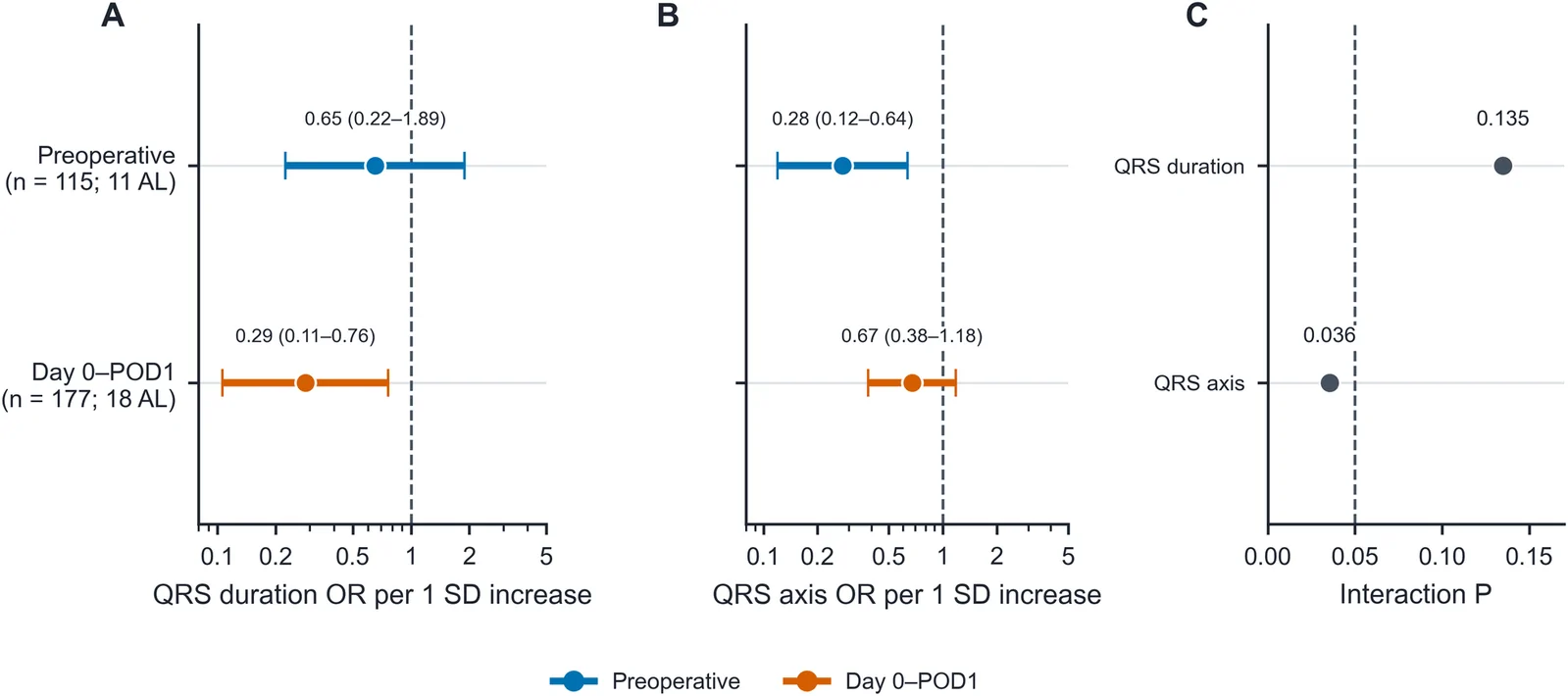
Timing-stratified ECG associations with anastomotic leakage. **(A)** Standardized ORs and Wald 95% CIs for QRS duration and **(B)** frontal-plane QRS axis in the preoperative and combined day-0-to-postoperative-day-1 groups. The preoperative group included 115 participants and 11 AL events; the day-0-to-postoperative-day-1 group included 177 participants and 18 events. ORs represent a one-SD increase using the full-cohort scales of 16.708 ms for QRS duration and 37.488° for QRS axis. The dashed line marks OR = 1. **(C)** Grouped timing-interaction *P* values; the dashed line marks *P* = 0.05. Postoperative day 1 contributed 23 participants and one event within the combined group and was not modeled separately. AL, anastomotic leakage; CI, confidence interval; OR, odds ratio; POD1, postoperative day 1; SD, standard deviation.

As presented in Figure 3C, the grouped interaction *P* value was 0.036 for QRS axis, providing evidence that its association differed between the two periods. The QRS-duration interaction *P* value was 0.135, indicating insufficient evidence to establish timing heterogeneity for QRS duration. The different stratum-specific QRS-duration estimates are therefore descriptive and should not be interpreted as proof that the association differed between periods.

We further assessed the discriminatory pattern of the three models in the same 292 participants. The apparent AUCs were 0.686 for the clinical reference model, 0.718 for the ECG model, and 0.778 for the combined model, as displayed in Figure 4A. Under repeated cross-validation, the median AUC was 0.559 for the clinical reference model, 0.702 for the ECG model, and 0.690 for the combined model. Figure 4B shows the corresponding empirical fold intervals, and Table 3 provides the numerical results. The ECG-containing models showed numerically higher discrimination than the clinical reference, but these point estimates did not by themselves establish improved prediction. Full coefficients for all three models are reported in Supplementary Table S2.

**Figure 4:**
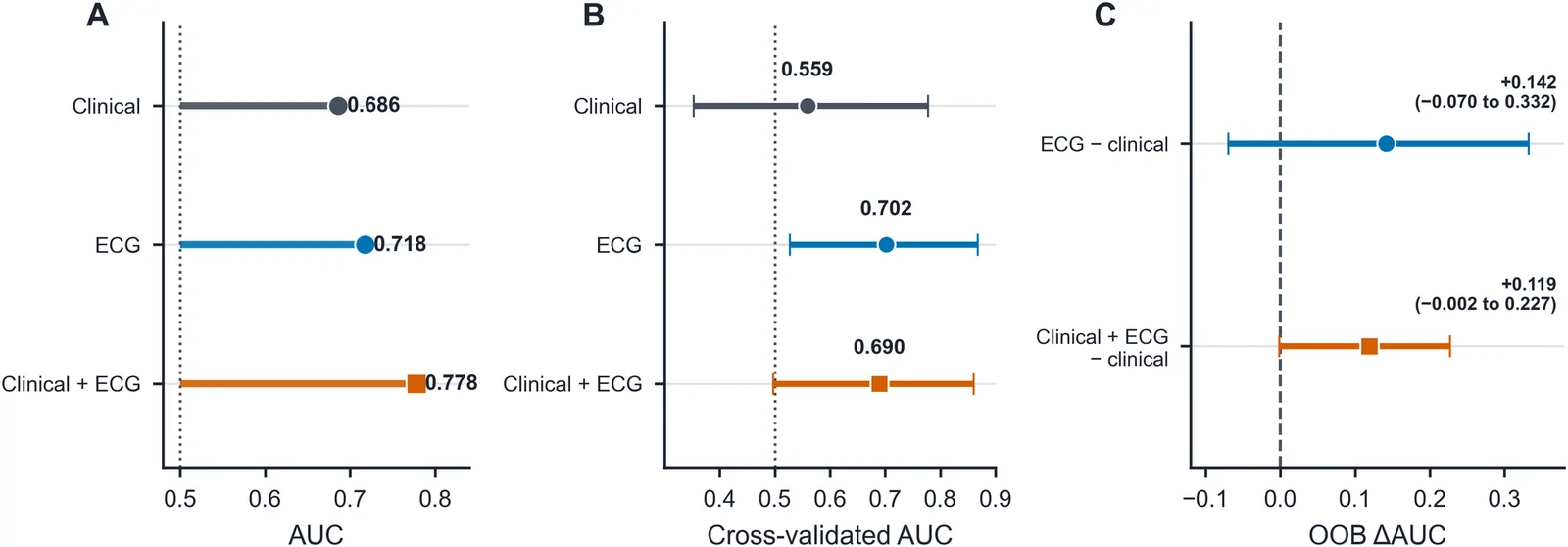
AUC comparison across clinical and ECG models. **(A)** Apparent AUCs. **(B)** Median AUCs from 100 repeats of stratified five-fold cross-validation; bars show the 2.5th–97.5th percentiles of 500 held-out-fold AUCs. **(C)** Patient-level out-of-bag bootstrap *Δ*AUCs (1,000 resamples): ECG minus clinical is non-nested, whereas clinical plus ECG minus clinical is incremental. Points show means and bars percentile 95% CIs. All models used 292 participants and 29 AL events. AUC, area under the receiver operating characteristic curve; CI, confidence interval; CV, cross-validation; ECG, electrocardiogram; OOB, out-of-bag.

**Table 3:** Model discrimination in the same analysis cohort.

Model	Predictors	Apparent AUC	Median CV AUC (empirical fold interval)	OOB ΔAUC vs clinical (95% CI)
Clinical reference	Seven clinical variables	0.686	0.559 (0.352–0.777)	Reference
ECG model	QRS duration and QRS axis	0.718	0.702 (0.527–0.868)	0.142 (−0.070–0.332)
Clinical+ECG	Seven clinical variables plus QRS duration and QRS axis	0.778	0.690 (0.497–0.860)	0.119 (−0.002–0.227)

All models included the same 292 participants and 29 AL events. CV used 100 repeats of stratified five-fold splitting. Empirical fold intervals are the 2.5th–97.5th percentiles across 500 dependent held-out folds and are not confidence intervals. Model-comparison intervals are percentile 95% CIs from 1,000 patient-level OOB bootstrap resamples. The ECG-minus-clinical contrast is non-nested; the clinical-plus-ECG-minus-clinical contrast is the incremental comparison. AUC, area under the receiver operating characteristic curve; CI, confidence interval; CV, cross-validation; ECG, electrocardiogram; OOB, out-of-bag.

Patient-level out-of-bag bootstrap comparisons produced positive point estimates versus the clinical reference: delta AUC was 0.142 (95% CI, -0.070 to 0.332) for the ECG model and 0.119 (95% CI, −0.002 to 0.227) for the combined model. Figure 4C shows both paired contrasts against a zero-difference reference. Both 95% CIs included zero. Therefore, the available cohort did not provide definitive evidence of incremental discrimination, and the model comparisons were interpreted as supportive but uncertain.

## 4 Discussion

This hypothesis-generating study identified associations between two routinely reported ECG measurements and AL after esophagectomy. Across the pooled perioperative window, patients with AL had a shorter QRS duration and a more leftward frontal-plane QRS axis. Both measurements remained associated after mutual adjustment and in the sensitivity model that additionally adjusted for seven clinical variables. The observed gradient across the number of ECG criteria made the direction of the associations readily visible. The ECG-containing models also showed numerically higher discrimination than the clinical reference model, although the delta-AUC confidence intervals included zero. Taken together, the findings support a reproducible association within this cohort while requiring prospective confirmation.

The pooled analysis and the timing analysis answer related but different questions. The pooled analysis reflects the association encountered when one perioperative ECG within the routine-care window is available for each patient. The timing analysis then separates day −1 recordings, which clearly precede surgery, from recordings assigned to the surgery day or postoperative day 1. The day −1 ECG can be interpreted as a baseline characteristic associated with subsequent AL. The day-0-to-postoperative-day-1 group is better interpreted as a perioperative or early monitoring signal because surgery-day clock times were unavailable and the measurements may incorporate both pre-existing susceptibility and consequences of surgery. The pooled findings should not be described uniformly as preoperative prediction.

The timing-stratified estimates require different levels of interpretation. For frontal-plane QRS axis, the axis-by-timing interaction supported a difference between periods, with the stronger association observed preoperatively. For QRS duration, the estimate was stronger in the day-0-to-postoperative-day-1 group, but the interaction *P* value of 0.135 provided insufficient evidence that the association truly differed by timing. The QRS-duration strata should therefore be treated as descriptive rather than as proof of a time-specific or complementary effect. Moreover, these analyses compared different patients' index ECGs across timing groups rather than serial ECGs within the same patients, so the data do not demonstrate postoperative QRS shortening in individuals.

Operation duration illustrates why temporal ordering matters. Longer operations were observed among patients who developed AL. For a preoperative ECG, operation duration occurs later and cannot cause the ECG measurement, although it may identify a surgical pathway that contributes to AL. For an ECG obtained on the surgery day or postoperative day 1, operation duration precedes the measurement and could influence it through anesthetic exposure, fluid administration, temperature, metabolic state, or operative stress. It may also be related independently to AL. Consequently, operation duration cannot be assigned the same causal role across ECG time points, and the early postoperative QRS association cannot be assumed to represent physiological information wholly independent of the operation itself. This reinforces the need for prospective studies with fixed acquisition times and richer intraoperative covariates.

There is currently no established biological mechanism linking shorter QRS duration or a leftward QRS axis to AL. The two measurements have different determinants, making a single causal chain unlikely on the present evidence. Frontal QRS axis varies with cardiac orientation, thoracic anatomy, body build, and diaphragmatic position (15–18). Separately, esophagectomy and gastric-conduit reconstruction create anatomical constraints in which conduit route, anastomotic tension, thoracic inlet space, compression, and perfusion may influence leakage (8, 32). One possibility is therefore that a preoperative axis difference indexes body or mediastinal geometry that is also relevant to conduit positioning. This does not mean that electrical axis measures anastomotic pressure or causes leakage. QRS duration around surgery could additionally be influenced by loading conditions, electrolyte and acid-base balance, inflammation, autonomic state (33), temperature, perfusion, or operative stress. These pathways remain untested hypotheses because the study did not measure muscle mass, autonomic activity, conduit tension, tissue perfusion, serial inflammatory physiology, or the proposed geometric intermediates.

The model analyses provided a quantitative check on the strength of the ECG associations. The clinical reference model supplied a benchmark based on conventional patient and procedural characteristics, while the combined model evaluated the QRS coefficients after clinical adjustment and quantified the change in discrimination when ECG measurements were added on the same analysis set. The positive delta-AUC point estimates were compatible with useful ECG information, but both bootstrap confidence intervals crossed zero. This does not establish equivalence or negate the association; it indicates that the magnitude of any incremental discrimination was uncertain in a cohort with only 29 events. The lower cross-validated AUC of the combined model relative to the simpler ECG model further illustrates the instability of adding predictors in a small event set. Accordingly, these models should not be interpreted as a finalized prediction system.

The findings nevertheless have practical research relevance. QRS duration and QRS axis are automatically generated in standard ECG reports, impose no additional blood draw, and are familiar to clinicians. If the associations are prospectively reproduced, both measurements could be evaluated at fixed preoperative and postoperative time points alongside established clinical observations and laboratory trends. Such use would complement rather than replace clinical assessment, biomarkers, imaging, or direct evaluation of a suspected leak. The same-cohort cut points should not be used to reduce monitoring, accelerate feeding, trigger computed tomography, or alter treatment. The next step is to test continuous ECG measurements at prespecified times and determine whether they add reliable information in independent cohorts.

Several design features strengthen the interpretation of the current findings. The study focused on quantitative measurements already available in standard ECG reports, retained continuous variables in the association models, and presented coefficients, ORs, and confidence intervals rather than relying only on a dichotomous risk grouping. Timing-specific estimates separated preoperative from day-0-to-postoperative-day-1 observations and made their different temporal interpretations explicit. All three discrimination models were evaluated in the same participants, and repeated cross-validation and paired patient-level bootstrap comparisons exposed performance variability rather than reporting apparent AUCs alone.

The study also has important limitations. It was retrospective and single-center, and only 20.3% of the source surgical cohort had an eligible perioperative ECG. ECG acquisition was not protocol-driven, and the observed differences in age, smoking history, and anastomosis location between patients with and without an eligible ECG indicate potential selection bias. The export did not retain manufacturer, device model, algorithm version, detailed conduction labels, surgery-day clock times, or dates of first AL suspicion and diagnosis; measurement heterogeneity and temporal ambiguity therefore remain possible. QRS duration and axis were selected after same-cohort screening without multiplicity adjustment, and feature selection was not repeated within resampling. Only 29 AL events were available, limiting the precision and stability of timing-specific estimates and of the seven- and nine-variable comparison models. Histological subtype, tumor etiology, stage, and grade were not completely available in the analytic dataset and could not be incorporated reliably. The two descriptive cut points were derived and evaluated in the same cohort and are therefore optimistic. Other relevant inflammatory, nutritional, neoadjuvant-treatment, perfusion, and intraoperative variables were unavailable, and the proposed biological pathways were not directly measured. These limitations require independent validation in prospectively timed, multicenter cohorts with complete oncological and operative characterization before clinical use.

## 5 Conclusion

Shorter QRS duration and a more leftward frontal-plane QRS axis were associated with AL after esophagectomy in this hypothesis-generating cohort. The QRS-axis association showed evidence of timing heterogeneity, whereas QRS-duration estimates differed descriptively without sufficient interaction evidence to establish a timing difference. ECG-containing models showed numerically higher discrimination than the clinical reference, but incremental value remained uncertain because the delta-AUC confidence intervals included zero. Routine QRS measurements are potential signals, not established predictors. Prospective multicenter studies should use standardized ECG acquisition times and incorporate histological subtype, tumor stage, grade, and relevant perioperative factors.

## Data Availability

The data analyzed in this study is subject to the following licenses/restrictions. The dataset contains patient-level clinical and electrocardiographic information that may permit participant identification. Public disclosure was not authorized under the study’s ethics approval. Access is therefore restricted to protect participant privacy and confidentiality. Requests for access may be directed to the corresponding author and will be considered by Shanghai Chest Hospital for qualified research purposes, subject to institutional review, applicable ethics approval, and a data-use agreement. Requests to access these datasets should be directed to BH, bin_he@sjtu.edu.cn.
