# Supplementary material for: Shorter perioperative QRS duration and a leftward frontal-plane QRS axis are associated with anastomotic leakage after esophagectomy: a hypothesis-generating study
Source: Front Med (Lausanne). 2026 Sep 29;13:1942699. doi: 10.3389/fmed.2026.1942699 (PMC13658592; doi:10.3389/fmed.2026.1942699)
Supplement: Supplementary file 1 [file Table1.docx]

Supplementary Material

# 1 Supplementary Tables

Supplementary Table S1. Comparison of source-cohort patients with and without an eligible early perioperative ECG

| **Variable** | **With ECG (n=292)** | **Without ECG (n=1149)** | **P value** |
| --- | --- | --- | --- |
| Age, years | 69.0 [63.0–74.2] | 67.0 [60.0–72.0] | <0.001 |
| Male, n (%) | 250 (85.6) | 987 (85.9) | 0.925 |
| BMI, kg/m² | 22.7 [20.5–24.8] | 22.6 [20.5–24.7] | 0.706 |
| Diabetes mellitus, n (%) | 44 (15.1) | 137 (11.9) | 0.166 |
| Current or former smoker, n (%) | 51 (17.5) | 145 (12.6) | 0.035 |
| Operative duration, min | 258.5 [222.8–302.2] | 258.0 [225.0–305.0] | 0.973 |
| Cervical anastomosis, n (%) | 186 (63.7) | 504 (43.9) | <0.001 |
| Anastomotic leakage, n (%) | 29 (9.9) | 132 (11.5) | 0.532 |

Patients with available ECG recordings differed from those without ECG in age, smoking history, and anastomosis location, indicating potential selection related to ECG availability. Continuous variables: median [IQR], Mann–Whitney U. Categorical: n (%), Fisher exact. ECG, electrocardiogram; BMI, body mass index.

Supplementary Table S2. Full standardized logistic-regression coefficients

| **Model** | **Term** | **Coefficient (SE)** | **OR (95% CI)** | **P value** |
| --- | --- | --- | --- | --- |
| ECG | Intercept | -2.567 (0.270) | 0.077 (0.045–0.130) | <0.001 |
| ECG | QRS duration | -0.974 (0.384) | 0.378 (0.178–0.801) | 0.011 |
| ECG | Frontal-plane QRS axis | -0.690 (0.225) | 0.501 (0.323–0.779) | 0.002 |
| Clinical reference | Intercept | -2.375 (0.225) | 0.093 (0.060–0.144) | <0.001 |
| Clinical reference | Age | 0.130 (0.210) | 1.138 (0.755–1.717) | 0.536 |
| Clinical reference | Male sex | 0.169 (0.229) | 1.184 (0.756–1.854) | 0.460 |
| Clinical reference | Body mass index | 0.348 (0.195) | 1.416 (0.965–2.077) | 0.075 |
| Clinical reference | Diabetes mellitus | 0.050 (0.187) | 1.051 (0.729–1.515) | 0.790 |
| Clinical reference | Current or former smoking | 0.275 (0.187) | 1.316 (0.912–1.899) | 0.142 |
| Clinical reference | Operation duration | 0.324 (0.186) | 1.383 (0.961–1.991) | 0.081 |
| Clinical reference | Cervical anastomosis | -0.389 (0.204) | 0.677 (0.455–1.010) | 0.056 |
| Clinical + ECG | Intercept | -2.786 (0.306) | 0.062 (0.034–0.112) | <0.001 |
| Clinical + ECG | Age | 0.141 (0.236) | 1.151 (0.725–1.828) | 0.550 |
| Clinical + ECG | Male sex | 0.247 (0.238) | 1.280 (0.803–2.042) | 0.300 |
| Clinical + ECG | Body mass index | 0.302 (0.211) | 1.353 (0.895–2.045) | 0.152 |
| Clinical + ECG | Diabetes mellitus | -0.011 (0.197) | 0.989 (0.673–1.454) | 0.955 |
| Clinical + ECG | Current or former smoking | 0.341 (0.201) | 1.406 (0.949–2.084) | 0.089 |
| Clinical + ECG | Operation duration | 0.423 (0.205) | 1.527 (1.021–2.282) | 0.039 |
| Clinical + ECG | Cervical anastomosis | -0.465 (0.217) | 0.628 (0.410–0.962) | 0.032 |
| Clinical + ECG | QRS duration | -1.024 (0.383) | 0.359 (0.170–0.761) | 0.007 |
| Clinical + ECG | Frontal-plane QRS axis | -0.722 (0.247) | 0.486 (0.299–0.789) | 0.004 |

All models included 292 participants and 29 AL events. Continuous and binary predictors were standardized within the model analysis set; ORs therefore represent a one-SD increase. Binary variables were coded 1 for male sex, diabetes mellitus, current or former smoking, and cervical anastomosis, respectively. The clinical reference model contained seven variables, and the combined model added QRS duration and frontal-plane QRS axis. AL, anastomotic leakage; CI, confidence interval; ECG, electrocardiogram; OR, odds ratio; SD, standard deviation; SE, standard error.

Supplementary Table S3. Screening results of routinely reported ECG variables

| **ECG variable** | **Association analysis result** | **P value** | **Retained** |
| --- | --- | --- | --- |
| Heart rate | No clear distributional difference by AL status | 0.239 | No |
| QRS duration | Lower values in patients with AL | 0.016 | Yes |
| Frontal-plane QRS axis | More leftward values in patients with AL | 0.006 | Yes |

The auditable analytic extract contained these three continuous ECG measurements. Results are from two-sided Mann-Whitney U tests in 292 participants, including 29 with AL. Screening was data-driven, P values were descriptive without multiplicity adjustment, and retained variables were selected and analyzed in the same cohort. AL, anastomotic leakage; ECG, electrocardiogram.
